# Streptococcus viridans Endocarditis Affecting All Four Valves

**DOI:** 10.7759/cureus.4635

**Published:** 2019-05-10

**Authors:** Manish Kumar, Emily J Anstadt, Nerea Lopetegui Lia, Misbahul H Siddiqi

**Affiliations:** 1 Internal Medicine, University of Connecticut Health Center, Farmington, USA; 2 Internal Medicine, Hartford Hospital, Hartford, USA

**Keywords:** hypertrophic obstructive cardiomyopathy, dental caries, viridans, native valve endocarditis, atrial septal defect (asd)

## Abstract

Infective endocarditis (IE) affecting all four valves is rare. We describe an interesting clinical scenario of a 47-year-old female, with an underlying, unrepaired atrial septal defect (ASD) and hypertrophic obstructive cardiomyopathy (HOCM), who presented with a five-week history of worsening lower extremity rash and New York Heart Association (NYHA) class IV symptoms. She was febrile to 101.3°F at the time of presentation. Examination revealed dental caries and track mark on hands. Her extremities demonstrated palpable purpura and pitting edema. Chest auscultation revealed bibasilar crackles and a grade III pan-systolic murmur, best heard over the apex, with radiation to the axilla. The blood gram stain resulted positive for gram-positive cocci in chains, prompting the initiation of ceftriaxone. Transthoracic echocardiography (TTE) did not reveal any new valvular regurgitation or vegetation. Transesophageal echocardiography (TEE) showed vegetation on all four valves and underlying ASD with HOCM. Blood cultures grew Streptococcus viridians. She had evidence of extensive septic emboli to the brain, lungs, spleen, and intestines. Given the extent of valvular involvement, intracranial hemorrhage, and tenuous hemodynamic status, a decision was taken to manage her conservatively followed by elective surgical management. She, however, went into cardiogenic shock further complicated by lower gastrointestinal bleed and passed away.

## Introduction

Infective endocarditis (IE) is a significant cause of morbidity and mortality. Endocarditis affecting all the four valves is extremely rare. We describe a case of pan-valvular endocarditis caused by Streptococcus viridans* *from dental caries in a patient with underlying hypertrophic obstructive cardiomyopathy (HOCM), unrepaired ostium primum atrial septal defect (ASD), and highlight the importance of early surgical intervention.

## Case presentation

A 47-year-old female presented with a five-week history of worsening purple, raised, painless, and non-pruritic lower extremity rash that started on her feet and progressed proximally. She reported progressive leg swelling and dyspnea with NYHA class IV symptoms. She denied chest pain or syncopal symptoms. There was no history of fever, chills/rigors, night sweats, arthralgia, or eye symptoms. Her travel history was negative.

The patient was febrile to 101.3°F at the time of presentation. Examination revealed conjunctival pallor, dental caries, track marks on the dorsal right hand, and bilateral lower extremity pitting edema. Her extremities demonstrated palpable purpura. Chest auscultation revealed bibasilar mid to late inspiratory fine crackles and a grade III pan-systolic murmur best heard over the apex with radiation to the axilla. Electrocardiogram (EKG) showed normal sinus rhythm, poor R-wave progression in anterolateral leads, and deep Q waves in V1 and V2.

Laboratory investigations were remarkable for leukocytosis of 12.6 k/uL, with 84.8% neutrophils, hemoglobin of 6.1 g/dL, elevated creatinine of 2.8 mmol/L, erythrocyte sedimentation rate (ESR) of 29 mm/Hr, and C-reactive protein of 7.01 mg/ dL. Troponin I was elevated at 0.42 ng/mL. Urine toxicology screen was negative, and urinalysis showed moderate protein and large blood.

**Figure 1:**
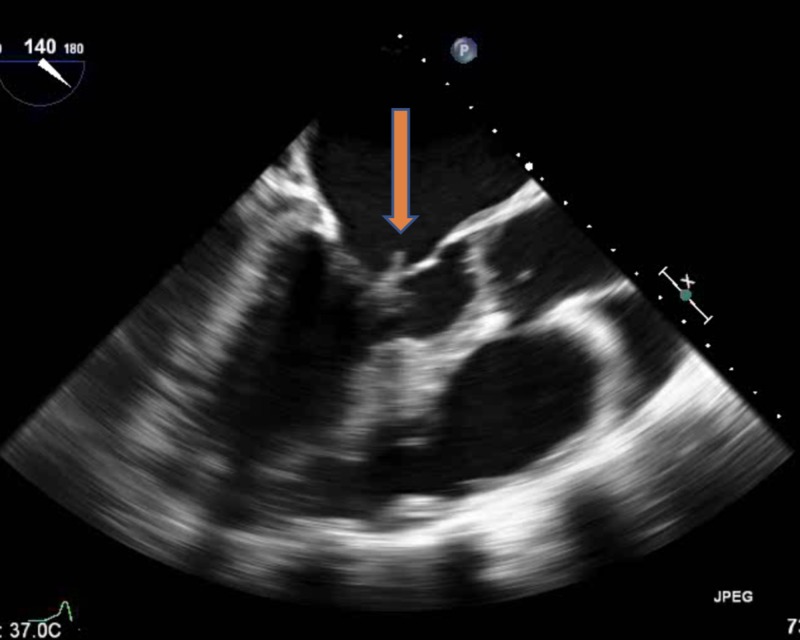
Mitral valve vegetation (arrow) on the anterior leaflet prolapsing into the left atrium during systole.

**Figure 2:**
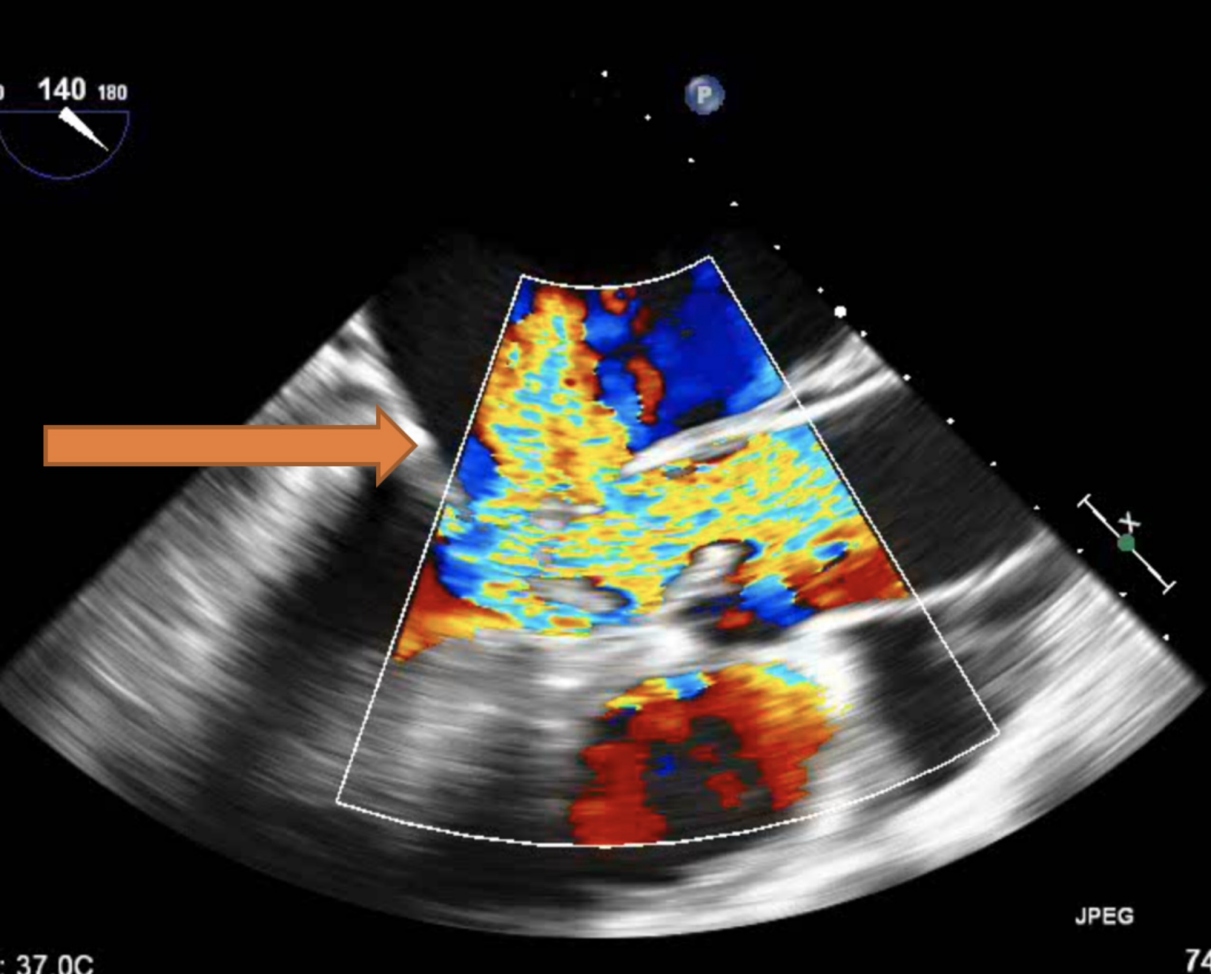
Arrow representing the mitral regurgitation jet caused by mitral valve vegetation.

**Figure 3:**
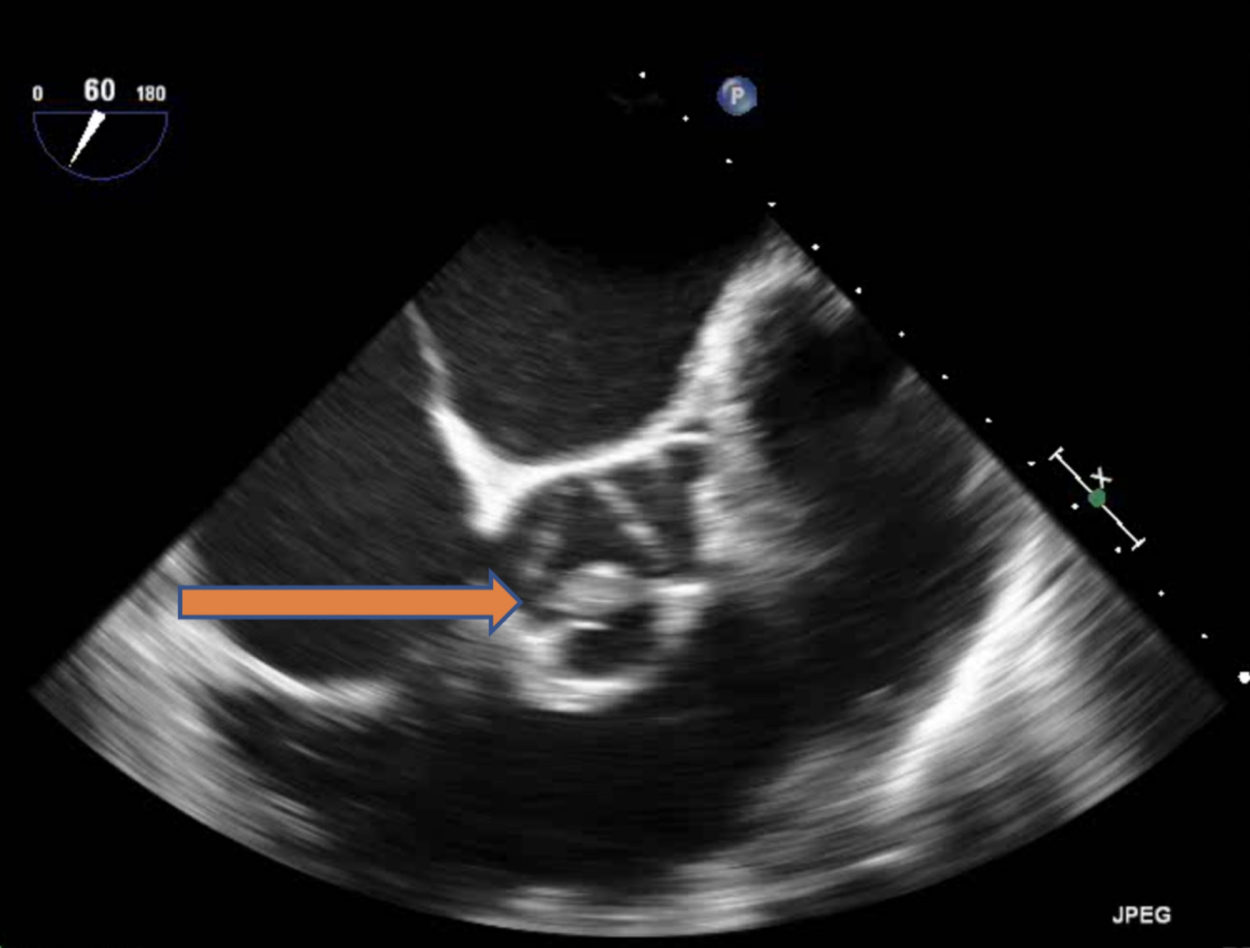
Aortic valve vegetation (arrow).

**Figure 4:**
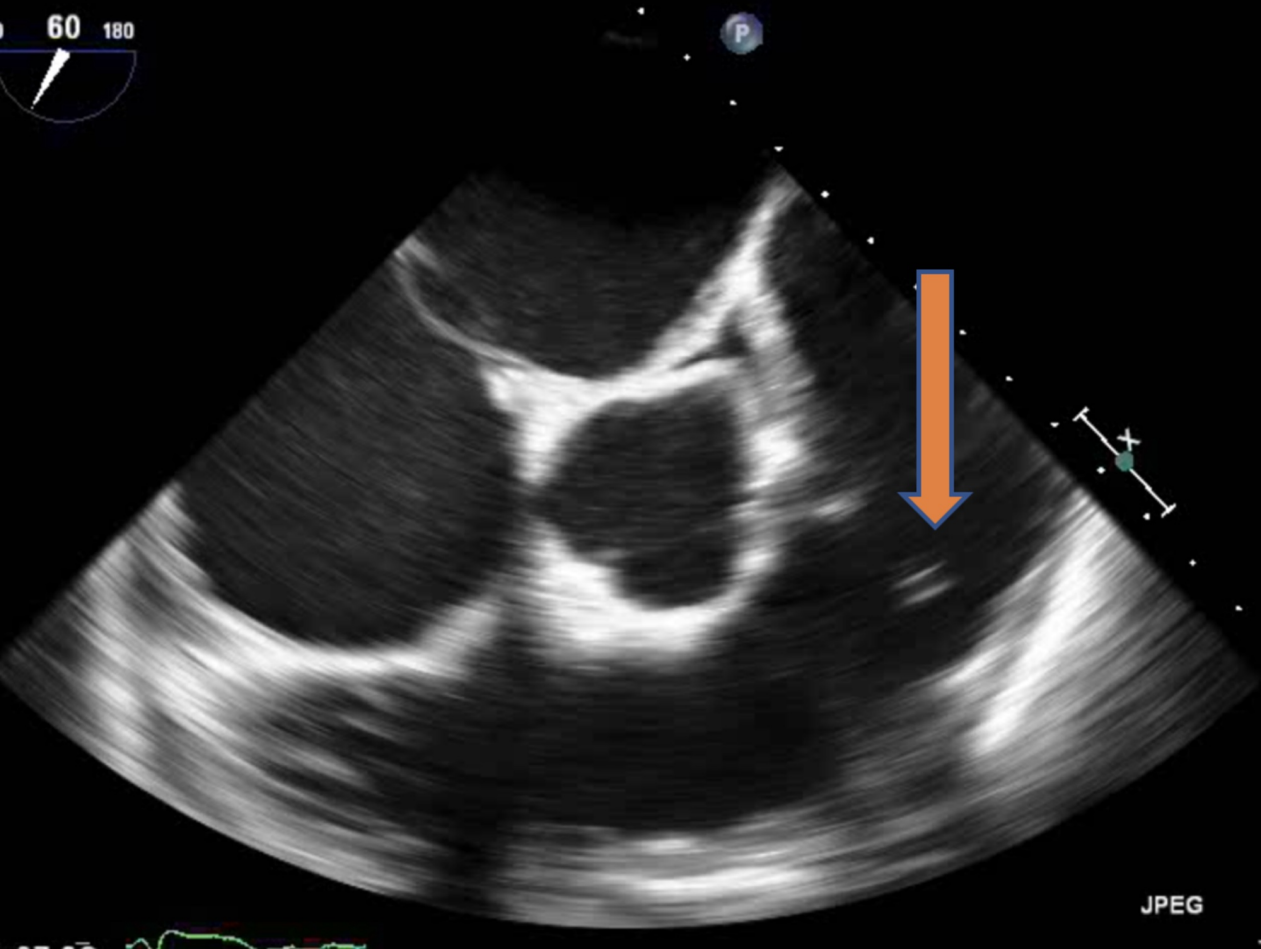
Pulmonic valve vegetation (arrow).

**Figure 5:**
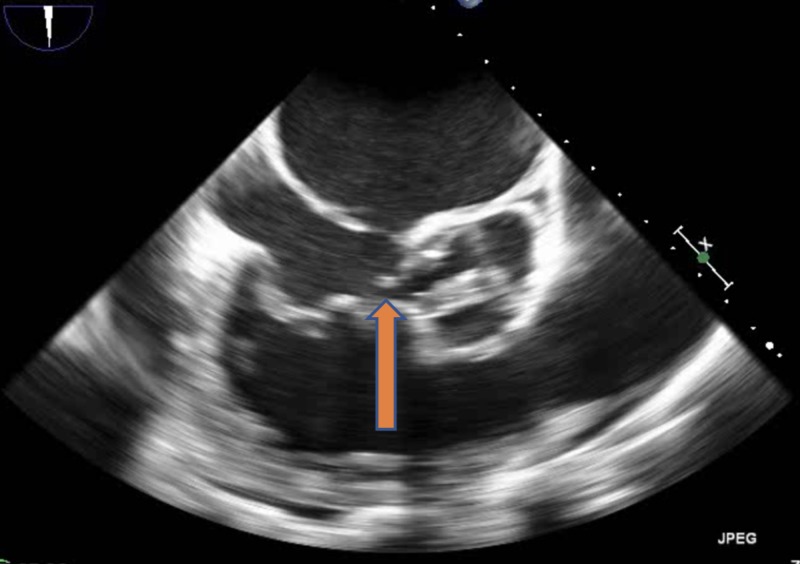
Tricuspid valve vegetation.

**Figure 6:**
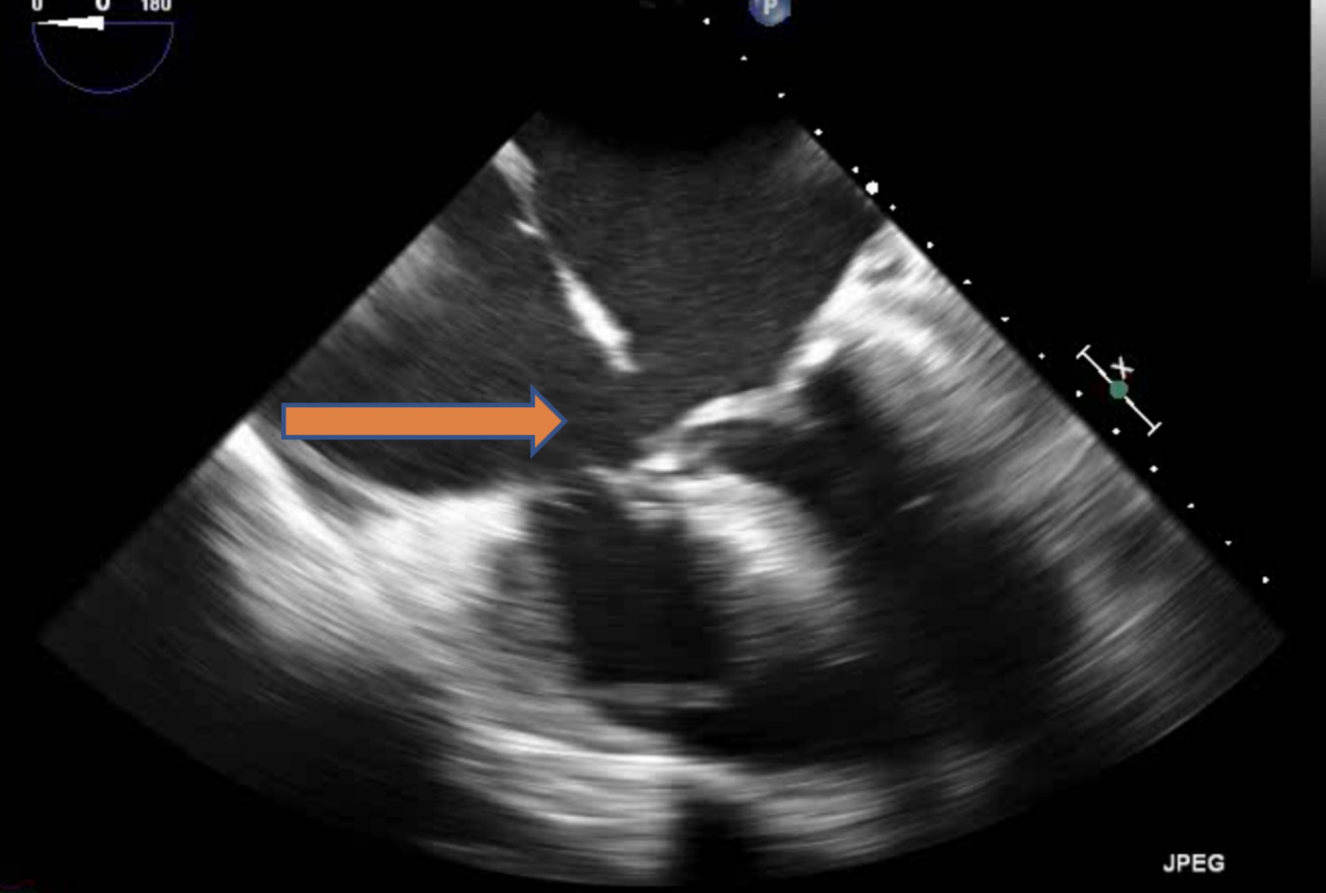
Atrial septal defect.

The blood culture gram stain resulted positive for gram-positive cocci in chains. She received vancomycin and ceftriaxone. The constellation of fever, positive blood cultures, and unrepaired ASD with associated dental caries raised the concern of sub-acute bacterial endocarditis (SABE). Transthoracic echocardiography (TTE) did not reveal any new valvular regurgitation or vegetation. SABE was strongly suspected and transesophageal echocardiography (TEE) was performed. It demonstrated a sizeable mobile echo density measuring 1.7 x 3.4 cm on the anterior mitral valve leaflet (Figure 1) causing mitral regurgitation (Figure 2) and left ventricular outflow tract obstruction, a mobile echo density on the aortic valve (Figure 3) causing moderate aortic regurgitation along with evidence of vegetation on the pulmonic valve (Figure 4), tricuspid valve leaflet (Figure 5), and ASD with a left to right shunt (Figure 6).

Blood cultures resulted in Streptococcus viridans. She had significant involvement on the left side. The right-sided vegetation might have been due to the embolic phenomenon from the left side because of her left to right shunt. During the hospitalization, she developed abdominal pain and lactic acidosis. Magnetic resonance imaging (MRI) of the abdomen showed splenic infarction due to septic emboli. Doppler revealed possible occlusion in the mid to distal superior mesenteric artery branches due to the septic embolus. MRI of the brain revealed multiple small septic embolic infarcts in bilateral cerebral hemispheres and discrete areas of microhemorrhage. Given the extent of her valvular involvement and the presence of intracranial hemorrhage, the decision was made initially to medically manage the right-sided valves with six weeks of antibiotics followed by early surgical management, as the perioperative risk was high. She, however, went into cardiogenic shock further complicated by lower gastrointestinal bleed and passed away.

## Discussion

Infective endocarditis (IE) is an infection of the endocardium with high morbidity and mortality without timely intervention. IE affecting all valves is extremely rare [1]. Patients with HOCM and other congenital heart diseases are at increased risk of developing IE as compared to the general population [2-3]. The surgical management of CHD usually reduces the incidence of IE [4]. Primary prevention in these patients becomes vital such as oral and dental hygiene and antibiotic prophylaxis. Antibiotic prophylaxis is usually limited to high-risk patients, such as individuals with a history of IE, prosthetic valve, and unrepaired cyanotic CHD undergoing dental procedure [5]. Surprisingly, left ventricular outflow tract obstruction in HOCM is not a risk factor, as similar rates of aortic and mitral valve involvement occur in patients with or without LVOT obstruction [6-7]. In patients with HOCM, the mitral valve is the most common valve affected due to elongated leaflets and continued erosion due to turbulent flow. Hence, these patients are at increased risk of persistent sepsis and heart failure [6,8]. Congenital heart disease usually predisposes to right-sided valvular involvement [7].

Endocarditis is usually managed medically with antibiotics. The three main indications for surgery are worsening heart failure or cardiogenic shock, uncontrolled infection, such as persistent positive blood cultures despite antibiotics or fungal endocarditis, and for the prevention of embolic phenomenon such as in cases of vegetation size >30 mm, or vegetation size more than 10 mm with an embolic event [8], or vegetation >20 mm with septic pulmonary emboli for right-sided valves [9]. Right-sided endocarditis is usually managed medically with antibiotics due to its higher recurrence rate especially in patients with ongoing drug abuse [9]. Surgical management has been proven to reduce mortality especially in patients having severe heart failure (NYHA III and IV) [5]. Earlier surgical intervention in patients with underlying unrepaired congenital lesions with superimposed endocarditis is advised before they develop worsening heart failure and hemodynamic instability, as these patients already have a limited cardiac reserve.

## Conclusions

Patients with HOCM and other congenital heart diseases are at increased risk of developing IE as compared to the general population. Earlier surgical intervention in patients with underlying unrepaired congenital lesions with superimposed endocarditis is advised before they develop worsening heart failure, as these patients already have a limited cardiac reserve.
